# Explaining Geographic Gradients in Winter Selection of Landscapes by Boreal Caribou with Implications under Global Changes in Eastern Canada

**DOI:** 10.1371/journal.pone.0078510

**Published:** 2013-10-23

**Authors:** Julien Beguin, Eliot J. B. McIntire, Daniel Fortin, Steven G. Cumming, Frédéric Raulier, Pierre Racine, Claude Dussault

**Affiliations:** 1 Département des sciences du bois et de la forêt and Centre d'étude de la forêt, Université Laval, Québec, Québec, Canada; 2 Pacific Forestry Centre, Canadian Forest Service, Natural Resources Canada, Victoria, British Coloumbia, Canada; 3 Département de biologie and Centre d'étude de la forêt, Université Laval, Québec, Québec, Canada; 4 Direction de l’expertise Énergie-Faune-Forêts-Mines-Territoire du Saguenay–Lac-Saint-Jean, Ministère des Ressources naturelles et de la faune, Joncquière, Québec, Canada; University of Alberta, Canada

## Abstract

Many animal species exhibit broad-scale latitudinal or longitudinal gradients in their response to biotic and abiotic components of their habitat. Although knowing the underlying mechanism of these patterns can be critical to the development of sound measures for the preservation or recovery of endangered species, few studies have yet identified which processes drive the existence of geographical gradients in habitat selection. Using extensive spatial data of broad latitudinal and longitudinal extent, we tested three hypotheses that could explain the presence of geographical gradients in landscape selection of the endangered boreal woodland caribou (*Rangifer tarandus caribou*) during winter in Eastern Canadian boreal forests: 1) climate-driven selection, which postulates that geographic gradients are surrogates for climatic gradients; 2) road-driven selection, which proposes that boreal caribou adjust their selection for certain habitat classes as a function of proximity to roads; and 3) an additive effect of both roads and climate. Our data strongly supported road-driven selection over climate influences. Thus, direct human alteration of landscapes drives boreal caribou distribution and should likely remain so until the climate changes sufficiently from present conditions. Boreal caribou avoided logged areas two-fold more strongly than burnt areas. Limiting the spread of road networks and accounting for the uneven impact of logging compared to wildfire should therefore be integral parts of any habitat management plan and conservation measures within the range of the endangered boreal caribou. The use of hierarchical spatial models allowed us to explore the distribution of spatially-structured errors in our models, which in turn provided valuable insights for generating alternative hypotheses about processes responsible for boreal caribou distribution.

## Introduction

The actual magnitude of global change that can be attributed to climate change and human-induced alterations of landscapes raises concerns about the adaptive abilities of many species to persist in this fast-changing world [1]. This view is supported by high current extinction rates for amphibians, birds, and mammals that are comparable to the rates prevailing during the last Big Five mass-extinction events, which have occurred over the past 443 million years [2]. Global warming and increasing anthropogenic disturbance are thought to be the two most important causes of current global change and the main causes of declines in biodiversity [3]. While both of these threats are anthropogenic [4], the ways of addressing species losses that are attributable to these two alternatives are quite different. Knowing which mechanism is most important in particular instances is vital if we are interested in putting into practice sound conservation measures for endangered species. 

In free-ranging animals, habitat selection is a fundamental behavioural process that structures their spatial distribution and influences population dynamics [5]. Fortin et al. [6] recently showed that the strength of selection (i.e., the relative probability of occurrence) for certain habitats varies along broad geographic gradients such as latitude and longitude, suggesting that large-scale processes may modulate the way in which animals respond locally to their environment. This result adds to previous findings that many mammal species exhibit broad-scale gradients in their responses to the environment, for example, through increasing body mass with latitude as predicted by Bergmann’s rule [7,8] or through changes in the strength of density-dependence relationships along latitude and longitude [9]. Change in habitat selection patterns along broad geographical gradients has both theoretical and practical implications. On one hand, it leads one to ask which ecological processes are responsible for this pattern; on the other hand, it stresses the need for management and conservation strategies that account for regional specificities in species responses to their environment. Despite their relevance in the context of global change, few attempts have been made to clarify possible causes of latitudinal and longitudinal gradients in habitat selection by moving organisms [10]. Latitude and longitude could be proxy variables for key drivers of global change such as climate conditions (e.g., temperature and precipitation) or human-induced alterations of landscapes. Thus, understanding the biological basis for such broad geographical trends would allow us to effectively determine species management priorities. 

We considered forest-dwelling or boreal woodland caribou (*Rangifer tarandus caribou*), hereafter boreal caribou, as a case study because it is a wide-ranging endangered ecotype [11] for which broad geographical gradients in landscape selection have been previously reported [6]. Our study focused on the Canadian eastern boreal forest within the southern part of the continuous range of boreal caribou. The sensitivity of boreal caribou to human-induced disturbances (e.g., logging [12]; roads [13]; petroleum and natural-gas infrastructures [14]) is well-documented throughout its distributional range in Canada. Further, these disturbances are thought to be the main causes of range recession in eastern North America [15–18]. Consequently, current conservation efforts are mainly oriented towards lowering human pressure on boreal landscapes [17]. Contrary to the situation in the western provinces of Canada where several boreal caribou populations are geographically isolated and the rate of anthropogenic disturbances is high within population ranges [19], the eastern boreal forest of Québec still contains large tracts of intact boreal forest, especially further north. In the context of lowering the human footprint, it is possible that climate plays an important role in the spatial distribution of boreal caribou. However, the role of climate in determining the large-scale distribution of, and landscape selection by boreal caribou has not been established. Climate can influence caribou through its direct and indirect effects on snow conditions, on forage accessibility and abundance, on levels of insect harassment, and on competition and predator-prey interactions [20]. Climate is known to influence the seasonal spatial distribution of barren-ground migratory caribou (*R*. *t. groenlandicus*) [21]. As the winter ranges of these two neighbouring ecotypes partially overlap in eastern Canada [22], boreal caribou also may respond to climatic gradients. The relative effects of climate and disturbance, or their additive effects on boreal caribou distribution have yet to be quantified. Disentangling the underlying processes from observed spatial patterns [23,24] can be challenging when spatial patterns originate from several processes that act in concert or when multiple causal processes have confounding spatial signatures at a given observational scale [25]. These issues are recurrent in macroecology and global change biology where, for example, the spatial distribution of climatic variables at low resolution often correlates with gradients in landscape alterations [26], making it unclear if climate alone, land-use patterns alone, or their additive effects drive species distributions at a broad spatial scale. Fortin et al. [6] reported residual spatial and geographic gradients, but it was not clear whether these gradients resulted from broad-scale effects of roads or they emerged from spatial variation in climate. 

Our study objectives were thus threefold. First, we tested three alternative *a priori* hypotheses regarding the possible causes of geographic gradients on caribou habitat selection: climate-driven selection, road-driven selection, and selection that is driven by an additive effect of climate and roads. The hypothesis of climate-driven selection assumes that previously known geographic gradients are a surrogate for climatic gradients in temperature and precipitation and that selection of certain habitats varies along climatic zones. The road-driven selection hypothesis reflects a trade-off between security and nutrition in which the boreal caribou adjusts its selection of certain habitat classes as a function of distance to roads [13,27]. The additive hypothesis considers that both mechanisms act in concert. Second, we offer a comparative evaluation of the impacts of logging and wildfire on boreal caribou spatial distribution to inform future conservation and ecosystem-based management strategies in the eastern boreal forest. Last, in exploring spatially structured errors in our models, we provide further testable hypotheses regarding alternative ecological processes involved in the broad-scale spatial distribution of boreal caribou. 

## Materials and Methods

### Ethics Statement

This study is restricted to visual observations of snow track networks of boreal caribou and, therefore, excludes any animal handling or invasive experiments. The study thus adheres to the “Canada guidelines for the Use of Animals in Research,” and to the legal requirements of Canada (http://www.ccac.ca/). All observations and surveys were conducted on public lands under the supervision of the Ministère des Ressources Naturelles et de la Faune du Québec (MRNFQ). All permissions were obtained from both legal authorities and public land managers. 

### Study area

**Figure 1:**
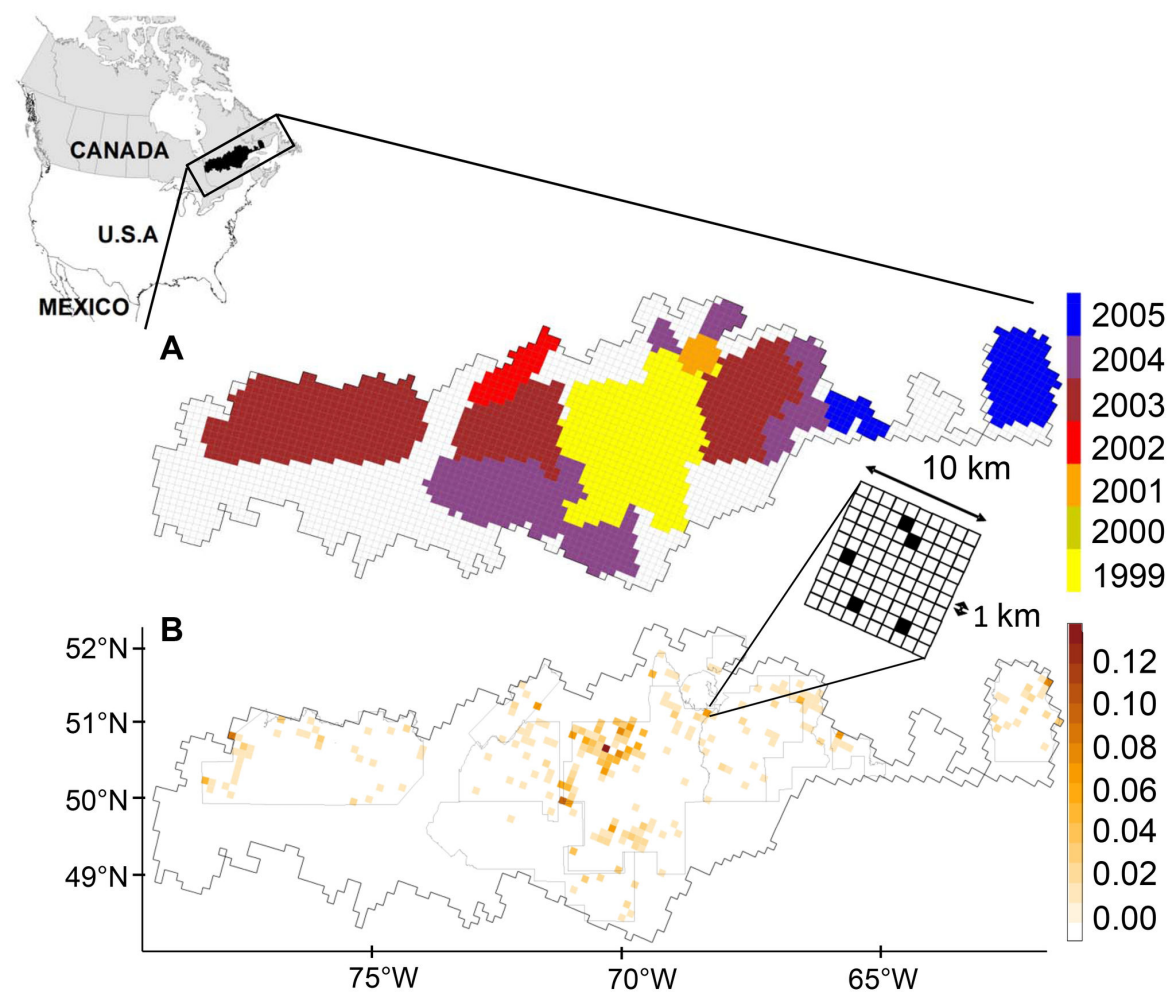
Study area showing A) the spatio-temporal design of aerial-survey blocks for the presence/absence of boreal caribou track networks; B) the proportion of intensive caribou track networks in each 100 km^2^ cell. Limits of inventory blocks are depicted by cells of different colours in A and by light grey contour lines in B. Uncoloured cells in A are located outside inventory blocks and are left unmonitored for the presence/absence of caribou tracks. White cells located within inventory blocks in B are monitored but correspond to the absence of caribou tracks.

The study region (~237 500 km^2^) was located in the coniferous boreal forest of eastern Canada (Figure 1), within the southern range of forest-dwelling caribou in the Province of Québec. The study area covered a wide geographic extent, ranging from 1300 km in longitude to 450 km in latitude. The area exhibited a high degree of variation in mean annual temperature (-4.0°C to 2.3°C), which is fairly representative of the mean annual temperatures observed across the natural range of boreal caribou (-7.1°C to 3.9°C, [28]). The percentage of the landscape that has been affected by anthropogenic disturbance in our study region (from 12 to 51 %) was comparable to that observed in many regions of Ontario (1 - 36 %) and Manitoba (3 - 26 %), but much lower than what has been witnessed in the western provinces of Alberta (21 - 95 %) and British Columbia (57 - 86 %) [19]. The area also exhibited high variability in road density from north to south (0 to 5.1 km/km^2^; Table 1). Vegetation was dominated by coniferous stands in which black spruce (*Picea mariana* (Miller) BSP), balsam fir (*Abies balsamea* (L.) Miller), and jack pine (*Pinus banksiana* Lambert) are the most frequently encountered canopy species (Table 1). White or paper birch (*Betula papyrifera* Marshall) and trembling aspen (*Populus tremuloides* Michaux) are found as companion species or in pure stands following recent disturbance. Open water bodies, wetlands, and bare land covered 11%, 10%, and 7% of the study area, respectively. Elevations ranged from 50 to 1000 m a.s.l., with plains and gentle hills (100-450 m) west of longitude 75°, and more rugged hills (300-1000 m) in the east. Wildfire and logging activities were the main disturbances throughout the region. The fire regime varies along a west-east gradient, with a shorter mean fire return interval in the west [29,30]. Extensive logging started in the early 1970’s in the southern part of the region and has since extended northwards, together with the road network. Contrary to barren-ground migratory caribou, the boreal caribou is sedentary and its spatial distribution ranges within the geographical limits of the Canadian boreal forest [18], from the eastern province of Newfoundland to the western province of British Columbia. In winter, boreal caribou live in small scattered and mobile groups of a few individuals. Caribou rely essentially on lichen in winter across its distribution range [12,31], but lichen is not limiting in some landscapes in Québec [12]. Boreal caribou has “vulnerable species” status in Québec. 

**Table 1:** Environmental variables that were used as predictors in our study.

Group of variables	Variables	Description	Minimum	Mean	Maximum
Habitat	**black spruce - jack pine** (**km^2^**) §	coniferous stands with basal area dominated by black spruce, followed by jack pine, or the converse	0	3.2	41.2
	**black spruce** (**km^2^**) §	coniferous stands with basal area dominated by black spruce	0	23.0	74.1
	**black spruce- balsam fir** (**km^2^**) §	coniferous stands with basal area dominated by black spruce, followed by balsam fir, or the converse	0	12.6	73.2
	**balsam fir** (**km^2^**) §	coniferous stands with basal area dominated by balsam fir	0	1.5	29.5
	**jack pine** (**km^2^**) §	coniferous stands with basal area dominated by jack pine	0	0.8	26.9
	**mixed resinous** (**km^2^**) §	mixed stands with basal area dominated by coniferous species, followed by deciduous species	0	2.5	30.8
	**lichen woodland (km^2^)**	coniferous stands of low density with abundant ground lichen	0	1.6	29.6
	**deciduous (km^2^)**	stands with basal area dominated by deciduous species (mainly birch and aspen)	0	1.4	39.0
	**mixed deciduous (km^2^)**	mixed stands with basal area dominated by deciduous species, followed by coniferous species	0	2.6	52.2
	**bare land dominated with lichen (km^2^)**	open dry sites with abundant ground lichen	0	2.9	42.1
	**water bodies (km^2^)**	lakes and rivers	0.1	11.2	93.1
	**wetlands (km^2^)**	bogs and fens	0	10.0	83.1
Road	road density (km/km^2^)	total road length by area unit	0	0.5	5.1
	**mean distance to road (km)**	average distance to all types of roads	0	10.3	105.5
Disturbance	**logging (km^2^)**	areas recently logged, with actual maximum tree height < 7 m	0	13.5	82.5
	**wildfire (km^2^)**	areas recently burned, with actual maximum tree height < 7 m	0	5.5	80.2
Climate	**Normal annual mean temperature** (°C) ¶	interpolated mean annual temperature (1970-1999)	-4.0	-0.8	2.3
	**Normal annual total precipitation** (**mm**) ¶	interpolated total annual precipitation (1970-1999)	740.9	976.1	1214.8

§ To avoid multicollinearity, all types of coniferous stands were reduced using principal component analysis: see Table S1.

¶ We also used winter normals of temperature and precipitation but no difference was observed, so we only retained annual variables.

Minimum, mean and maximum values of each explanatory variable, at the scale of 100 km^2^ grid cells, are presented for the entire region. Only variables that are shown in bold type were retained in the analysis of model comparisons (i.e., the variable “road density” was discarded because of its high correlation with the variable “logging,” i.e., *r* = 0.85).

### Caribou and environmental data

Our observational units were presences and absences of intensive caribou snow-track networks that had been observed during the winter season. A caribou snow-track network (hereinafter called caribou tracks) delineates an area that is used briefly and intensively in winter by a small group of caribou for foraging or shelter. These caribou tracks are thus closely related to the biological needs of boreal caribou in winter, a critical season for temperate ungulates (see 6 and references therein]). Caribou tracks were sampled from intensive fixed-wing aerial surveys, which were backed by helicopter spot-checks, conducted in the winters of 1999 through 2005 (for details, see 6,32). The surveys were conducted along transects within 11 spatial-temporal survey blocks (Figure 1). The probability of caribou track detection given presence was high (≥ 90 %), due to strong contrasts between caribou track and adjacent undisturbed snow surfaces [32]. Given the high detectability of caribou tracks and the intensity of sampling efforts, false positives and false negatives were ignored in this analysis. 

We related the probability of occurrence of winter caribou tracks to environmental conditions using 18 covariates belonging to four different classes of variables: habitats, disturbances, roads, and climate (Table 1). These environmental covariates were obtained from up-to-date digital forest inventories [33] and interpolated monthly climate data [34]. Forest inventory data allowed us to distinguish between forested and non-forested polygons. Forested polygons were classified according to species composition, density, and height (Table 1). Non-forested polygons distinguished various land-types such as disturbances, wetlands and water bodies. For testing the role of climate surfaces in explaining geographical gradients in habitat selection, we used 30-year averages of mean temperature and precipitation calibrated from 1970 to 1999 (hereafter called normals). The rationale for this choice is as follows: 1) these variables are the main climatic inputs for global climate models (GCMs), making their use potentially relevant to assessing future direct impacts of climate change, if any, on boreal caribou distribution; 2) prediction errors that are associated with these primary variables are low [34]. In contrast, the prediction error of snow depth data in our study was high, so that variable was not included in our models. 3) Temperature and precipitation data serve as main inputs for calculating derived climatic indices, making correlations between climate indices and these primary variables very likely. Seasonal and annual climatic variables were highly correlated (*r* > 0.8) and we found no difference in the results when climate variables were measured annually or over winter months. Therefore, we only retained 30-year annual normals, which were more likely to interact with vegetation attributes than just the winter climatic variables. Given the size of the data set (> 5 GB), all spatial data were processed using the open-source spatial database system PostGis (http://postgis.refractions.net/). Spatial resolution of covariates varied from 8 ha for forest inventory attributes to ~100 km^2^ for climate data. All attributes were calculated as total areas or averages over a common 100 km^2^ grid, which was defined by the climate data (see Table 1). Hereafter, each elementary unit of this grid is referred to as a “cell" (see Figure 1). Each covariate was then normalised by subtracting the mean and dividing by the standard deviation. We calculated 14 land cover covariates from the forest inventory data as proportional areas of various types of forested and non-forested habitats. Of these, six variables referred to various types of coniferous forest (Table 1). To avoid multicollinearity, we performed principal component analysis (PCA) with a covariance matrix based on these 6 variables and retained sample scores of the two most significant orthogonal axes as model covariates (see Table S1). Variance inflation factors in the reduced data set were < 3 for all covariates, indicating that multicollinearity amongst the predictors was not an issue [35]. Caribou presence/absence data were calculated for a 1-km^2^ resolution sub-grid (see Figure 1). This resolution was roughly twice the mean of caribou track area (0.53 km^2^, SE ± 0.06 km^2^: see 32) and well below the size of the winter home range, which typically varies from about 100 to 700 km^2^ [36,37].

### Statistical analyses

We built our candidate models upon alternative *a priori* hypotheses where the presence of geographical gradients in landscape selection was a surrogate for: i) an effect of climate, alone or in interaction with covariates (models 5 and 8; Table 2); ii) an effect of distance to roads, alone or in interaction with covariates (models 4 and 7; Table 2); or iii) an additive effect of climate and distance to roads with possible interactions with covariates (model 9; Table 2). For completeness, we include several neutral models: intercept only, landcover only, geography and land cover only, and geography, landcover and interactions (models 1, 2, 3 and 6, respectively; Table 2). All candidate models had the same structure but differed in their design matrix $x_{i}^{'}$ (see Text S1). Let *n*_*i*_ be the number of 1-km^2^ sub-cells within the *i* ^th^ cell, and *Y*_*i*_ is the number of these sub-cells where the presence of caribou track is observed. We assumed *Y*_*i*_ to be a binomial random variable and have modelled the probability *p*_*i*_ of caribou track presence within cells using a logit link function and the following generic generalised linear mixed model (GLMM):

$$Y\sim \;Binomial(n_{i},\;p_{i}),$$

(1)

where *x* ^′^_*i*_ is the vector of standardised covariates of each candidate model for each cell *i*; *β* is the vector of parameters associated with environmental covariates to be estimated for each candidate model, where each *β*_*j*_ is assigned a vague prior such as$\beta_{j}\overset{iid}{\sim }N(mean=0;precision=0.001)$; *α*_*k*_ is a random intercept of the *k* ^*th*^ inventory block (*k* = 1,…, 11; see Figure 1) that accounts for a block-specific value for intercept*α*, where 

$\alpha_{k}\overset{iid}{\sim }N(mean=0;precision=\tau )$and the hyperparameter *τ* is assigned a vague prior such as *τ*~ *Gamma*(*shape* = 1; *scale* = 5*e*−05) . We verified that residual spatial autocorrelation (RSA) did not affect the model selection procedure (see Text S2 and Figure S1). In addition to model selection and hypothesis tests, we were also interested in making inferences on parameters, especially in comparing the impact of fire *vs* logging on boreal caribou space use. Indeed, disentangling these effects is of particular concern from a conservation and ecosystem-based management perspective. Inference on model parameters, however, is particularly sensitive to RSA (e.g., increasing type-I error) so we explicitly modelled spatial associations amongst cells by adding a spatial random effect, with a Matérn correlation function, to the simplest and top-ranked model that had been previously selected (see Text S3 and Figure S2). The addition of a spatial random effect to Equation 1 also allowed us exploring the spatial structure in model errors which can, in turn, inform us about important ecological processes not accounted for in our models. 

**Table 2:** Model comparisons for the different *a* *priori* hypotheses tested in this study.

**ID model**	**Hypothesis**	**Variables**	**pD**	**REP**	**DIC**
4	distance to road (= distroad)	land cover + distroad	20	85	1766
7	distroad x covar	land cover + distroad + distroad:logging + distroad: woodlichen + distroad:water	23	74	1766
6	geog x covar	land cover + X + Y + Y:logging + Y:lichen woodland + Y:water ¶	24	71	1769
9	distroad x covar + climate x covar	land cover + distroad + meanprec + meantemp + meantemp:logging + meantemp: lichen woodland + meantemp:water + distroad: lichen woodland + distroad:logging + distroad:water	28	61	1769
8	climate x covar	land cover + meanprec + meantemp + meantemp: logging + meantemp: lichen woodland + meantemp:water	24	71	1795
3	Geographic (= geog)	X + Y + land cover ¶	21	80	1800
5	climate	land cover + meanprec + meantemp	21	81	1801
2	land cover only	land cover §	19	89	1803
1	intercept only	intercept	9	201	2094

§ land cover = intercept + wildfire + logging + lichen woodland + bare land dominated by lichen +

water + wetlands + coniferous stands PC1 + coniferous stands PC2 + deciduous +

mixed deciduous (see Table 1 for a definition of variables)

¶ X and Y represent the geographic coordinates of centroids for each 100 km^2^ cell.

Geographic-driven selection is represented by model IDs 3 and 6, climate-driven selection by model IDs 5 and 8, and selection that is driven by distance to roads is represented by model IDs 4 and 7. Interaction effects between two variables are indicated by a colon (:). The hypothesis of an additive effect of climate and distance to roads is represented by model ID 9. Variable names: pD = the number of effective parameters; REP = the mean number of data points for each effective parameter; and DIC = Deviance Information Criteria. See Table 1 for a description of variables.

All models were fit in R v2.14.2 [38], using integrated nested Laplace approximation (INLA) within the R-INLA package [39], which is freely available at www.r-inla.org. 

## Results

As expected, distance to roads was positively correlated with latitude, whereas the mean temperature gradient was negatively correlated with latitude. Precipitation was positively correlated with longitude (Table 3). Based on non-spatial GLMMs with 100% of the data, the ΔDIC between model 6 (DIC = 1769) and models 2 and 3 (DICs ≥ 1800) was greater than 30 (Table 2), indicating that the existence of geographical trends in caribou selection of lichen woodland, logging areas and water bodies better explained the data than did the hypothesis of homogeneous selection of these habitats across the study region. We failed to detect any top-down climatic control on these geographical gradients, as the models with climate variables alone or in interaction with land cover (models 5 and 8; Table 2) had less support than did model 6, which contained only geographical gradients (ΔDIC > 30; Table 2). In addition, climate variables only marginally explained extra information that was not already explained by land cover and disturbance variables (ΔDIC ≤ 2 between models 5 and 2; Table 2). Overall, models that included distance to roads, alone or in interactions with other landcover types (models 4, 7, 9), and the model that included interactions between latitude and landcover types (model 6) were the most supported amongst all of the candidate models. Model 6 indicated geographical trends in caribou selection, but we do not discuss it further because of its lack of mechanistic explanation. Model 9, which included interaction terms between climate and landcover types, had lower support than models 4 and 7, both without climatic variables (ΔDIC ≥ 3; Table 2), which indicates that addition of climate variables did not improve the fit of models 4 and 7. The low contribution of climate, alone or in interaction with land cover, to the model fit is further seen in the low support of models 5 and 8 (Table 2). The model with a single effect of the mean distance to road (model 4) and model 7, with an interaction between distance to road and landcover types, were thus the best supported candidate models. This shows that the large-distance effect of roads, rather than climate, plays an important role in boreal caribou habitat selection and provides a mechanistic explanation for geographical trends that was observed in habitat selection patterns. We could not distinguish, however, whether model 4 or 7 had the better fit (ΔDIC ≤ 2; Table 2). For inference, we adopted a pragmatic view and retained a spatial version of model 4, as it was the simplest top-ranked model. Boreal caribou positively selected water bodies and areas that were located far from roads, whereas they avoided deciduous stands and disturbances that had been caused by fire and logging (Table 4). Logged areas were avoided twice as strongly as burnt areas, showing that boreal caribou did not respond to these two disturbances in the same manner (Table 4). Boreal caribou tended to select positively lichen woodland, but the parameter estimate for this variable also exhibited a greater sensitivity to specification of the spatial random effect (Figure S2). No selection pattern was detected for coniferous and mixed stands, wetland, or open habitat with lichen ground cover (Table 4). 

**Table 3:** Pearson product-moment correlations (r) amongst values of geographical UTM coordinates of cell centroids (X for longitude and Y for latitude), annual climatic normals of total precipitation and mean temperature, and mean distance to roads (**see** Table 1 **for variable descriptions**).

	X (km)	Y (km)	Total precipitation (mm)	Mean temperature (°C)	Distance to roads (km)
X (km)	1				
Y (km)	0.24	1			
Total precipitation (mm)	**0.84**	-0.19	1		
Mean temperature (°C)	-0.22	**-0.81**	-0.01	1	
Distance to roads (km)	0.26	**0.60**	0.01	**-0.69**	1

Values in bold highlight |*r|* values > 0.5.

**Table 4:** Posterior medians of parameter estimates (with 95% credible intervals) for a spatially explicit version of the simplest and top-ranked model (**see model 4 in** Table 2).

Variables	2.5%	50%	97.5%
Logging	-1.17	-0.76	-0.39
Wildfire	-0.61	-0.37	-0.14
Coniferous stands (PC 1)	-0.32	-0.06	0.21
Coniferous stands (PC 2)	-0.24	-0.05	0.14
Lichen woodland	-0.03	0.10	0.23
Deciduous	-1.18	-0.70	-0.10
Mixed deciduous	-0.32	0.04	0.34
Bare land dominated by lichen	-0.29	-0.13	0.03
Water	0.11	0.30	0.48
Wetlands	-0.25	-0.01	0.23
Mean distance to road	0.07	0.29	0.46

Variable descriptions are presented in Table 1.

**Figure 2:**
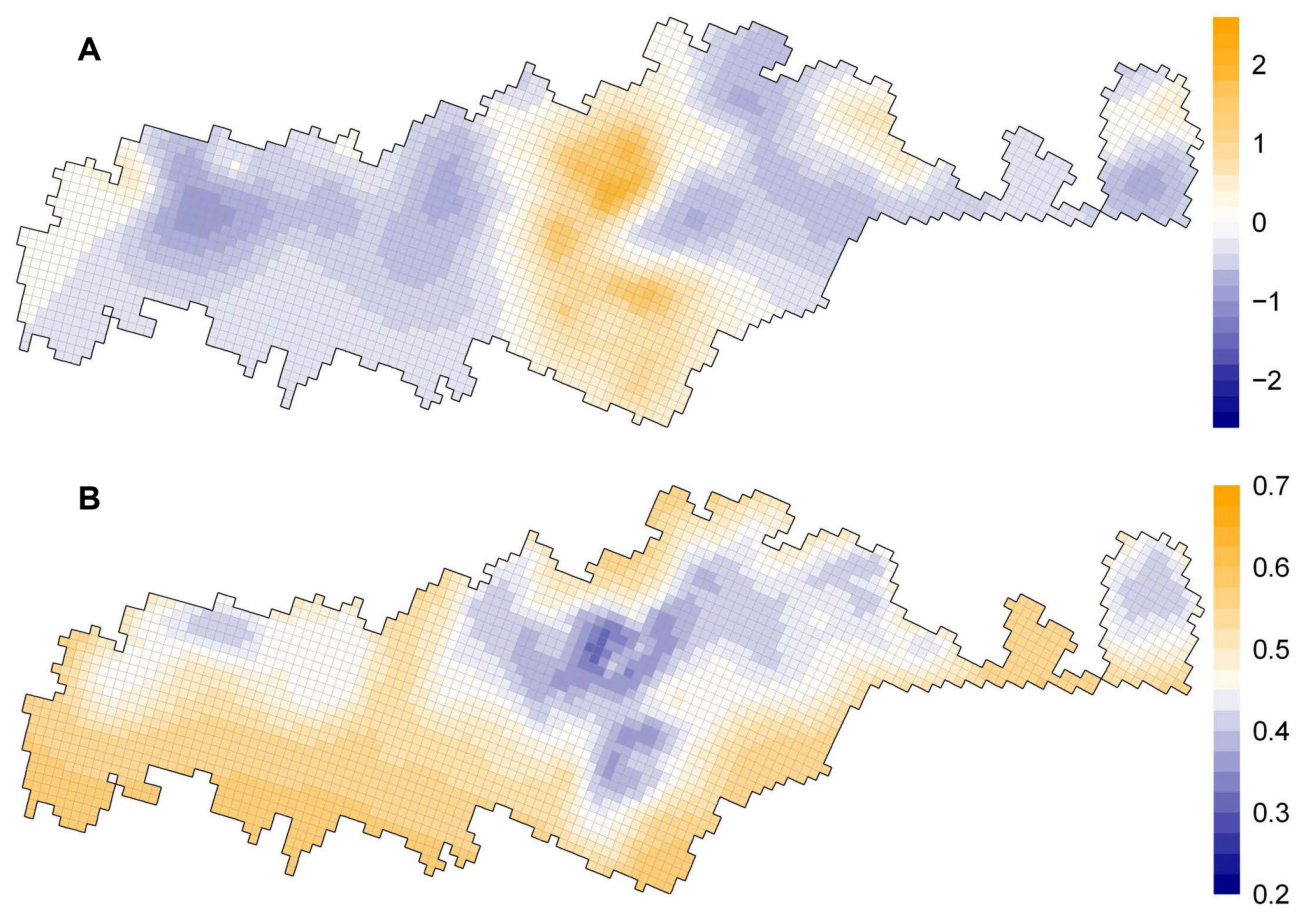
Map of the posterior mean (A) and posterior standard deviation (B) of the spatial random effect.

Mapping spatial random effects revealed strong latent spatial patterns at multiple scales (Figure 2A). At a coarse scale, favourable areas for boreal caribou are mainly concentrated in a large band that is centred on 70°W longitude and which extends from 48° to 52°N latitude. At an intermediate scale, suitable sub-regions emerged in the northern part of the study area, whereas unfavourable sub-regions appeared mainly in the central and southern parts of the study region. Within these sub-regions, finer-scale local variation was present. This multi-scale spatial pattern showed very low sensitivity to prior specification of the spatial random effect, as it was consistent for a wide range of values for the precision hyperparameter *τ*. As expected, uncertainty in the spatial random effect increased with increasing distance from the points at which data had been collected (Figure 2B). 

## Discussion

We rejected both the hypothesis of climate-driven winter selection of landscapes and the hypothesis of an additive effect of climate and distance to roads. Indeed, temperature and precipitation normals, which are the main inputs that are used in climate change scenarios, did not explain latitudinal trends observed in the winter selection of certain habitat classes by boreal caribou. Moreover, we failed to detect any effect of temperature and precipitation variables alone on the winter distribution of boreal caribou, once land cover and disturbance types were taken into account. These results contrast with evidence that temperature and precipitation play an important role in the winter distribution of the largest herd of migratory caribou in Québec [21], indicating that both ecotypes may not respond to the same environmental filters. From this difference, it can be anticipated that climate changes - specifically changes in mean temperature and total precipitation - should have more short-term direct impacts in winter on migratory caribou than on boreal caribou. The mechanisms for the apparent climate sensitivity of the migratory ecotype remain unknown but could be related to summer food limitation [40] – a rare or non-existent circumstance for the boreal ecotype. Although snow depth can influence fine-scale habitat selection of boreal caribou [41], the lack of precision of snow depth estimates at broad spatial scale prevented us to test reliably for the effect of snow depth on boreal caribou winter distribution and the possible relationship between snow depth and climatic variables. Further studies are needed to document the possible interrelationships amongst temperature, precipitation, snow conditions and the spatial distribution of boreal caribou. It is also important to assess if climate changes affect landscape selection by boreal caribou during the snow-free season. Our failure to detect any direct influence of seasonal or annual trends in mean temperature and total precipitation, however, does not imply that climate change will have no indirect effects on boreal caribou during winter. For example, climate change is expected to change the amount and spatial distribution of the area that is burned in the boreal forest of North America [42]. In turn, burnt areas will influence winter habitat selection, as shown in our study and by several others [43,44]. A full assessment of indirect effects of climate change on caribou spatial distribution will require further development of a dynamic multivariate simulation model that would explicitly model interdependencies over space and time amongst climate variables, disturbance regimes, vegetation types, and predator-prey interactions. 

The hypothesis of road-driven selection was well-supported by our data. Our findings were consistent with previous studies showing that the boreal caribou avoids roads [13,45]. We found an equivalent support for both hypotheses of dependence (model 7) and independence (model 4) in the selection of lichen woodland, water bodies, and logging areas with distance to roads. Our failure to discriminate these two alternatives could be related to the spatial resolution of our models, which might be too low for matching the finer spatial scale at which road distance-based selection might occur. MacArthur [46] hypothesised that in the northern hemisphere, species distributions are more likely to be limited by biotic interactions at the southern margin of their range and by abiotic factors at the northern edge. In this study, which occurs at the current southern range limits of boreal caribou, roads likely act as a surrogate for higher densities of alternate prey (e.g., moose) and predators (e.g., wolves) – the prevailing hypothesis for the decline of forest-dwelling caribou [18]. The predominance of human-induced disturbances over climate-induced effects in explaining distributional patterns of boreal caribou is in accordance with expectation. This is also consistent with a broad-scale niche analysis of this ecotype ([17], section 6.4). Hence, this study reinforces previous recommendations [15–17] that habitat conservation measures for boreal caribou should concentrate on minimising road density and logging areas at the landscape-level but also on reducing broad-scale expansion of road networks into the current ranges of boreal caribou. 

The management of boreal forest in the Province of Québec, including coniferous forests that are located within the southern range of boreal caribou, has recently changed paradigms from sustained yield towards the principles of ecosystem-based management [47,48]. One key principle of ecosystem-based forest management is to limit the rate of anthropogenic disturbances to conform to the historical ranges of ecosystem natural variability in ecosystem properties [49], such as species composition and age-class structure. In addition to these new guidelines, permanent and temporary forest blocks (hereafter named protection blocks) have been set aside from logging to improve the conservation of boreal caribou habitat within its actual range [50,51]. Logging activities are therefore permitted in the forest matrix that is located outside protection blocks but their rate, intensity, and spatial distribution are to be informed by historical patterns of natural disturbance regimes (e.g., fire and insect outbreaks). With this management strategy, it is assumed that the substitution of natural for anthropogenic disturbances of the same intensity and rate will maintain species diversity, together with the main ecological functions of ecosystems [52]. This assumes that logged and burnt areas are effectively equivalent. Our results show that they are not. Boreal caribou avoided logging areas twice as strongly as burnt areas, even after accounting for many other sources of variation. It follows that logging, at least as it is practised to date, is not equivalent to fire in terms of its effect on boreal caribou. A possible explanation for the uneven effect of logging and burnt areas on boreal caribou could originate from an increase of road densities that is associated with forest operations, which in turn increases predation risk because roads facilitate the displacement and search efficiency of predators [53,54]. Thus, substituting burnt areas with equivalent logging areas outside of protection blocks according to current ecosystem-based management guidelines is not likely to maintain the historical suitability of landscapes for boreal caribou. More importantly, wildfires are just as likely to occur within protection blocks as outside them, stressing the need for managing the forest matrix as a whole, including those forest areas that are not targeted for conservation, rather than focusing solely on isolated protection blocks. This study could not address how changes in landscape suitability will affect caribou population dynamics. However, a lower probability of occurrence often translates into decreases in population density [55], with evidence showing that logging, more than fire, negatively affects recruitment rate, a key demographic parameter of population rate-of-growth in caribou [17]. Ecosystem-based management of coniferous boreal forest, therefore, should account for the uneven effect of logging activities compared to that of burnt areas on wildlife species. This point is particularly sensitive given that the co-occurrence and cumulative effects of both disturbance types are expected to persist in the future, despite the delineation of protected areas, since fire control strategies are not one hundred percent efficient, especially in the far north. Further efforts are urgently needed to characterise the sensitivity of key species to various types of disturbances. Only then will a clearer picture help managers to develop efficient ecosystem-based management guidelines for the boreal forest that properly match conservation planning efforts for boreal caribou. 

The strong residual spatial pattern found in the best model reveals some phenomenon or process that we did not include in our models. There is a growing recognition amongst ecologists that the presence of residual spatial autocorrelation is not simply a statistical nuisance (e.g., increasing type-I error). Rather, it may also be an ecological opportunity [24], as it points to an important ecological process(es) that is (are) misspecified or lacking in the given hypothesis or model. While the accounting for spatial autocorrelation improved the validity of parameter inferences, it should be noted that our results are derived from observational data and, thus, are conditional to the alternative hypotheses that we tested. We argue that identifying patterns of spatial autocorrelation and uncovering their underlying causes are two important steps in understanding which unknown ecological driver(s) structures the spatial distribution of living organisms. The use of an *a posteriori* approach, where spatial dependency is modelled directly from the data, allows the structure of spatial autocorrelation patterns to be explored and provides strong direction for further hypotheses about the underlying causes of species distributions. The distribution of spatial random effects for boreal caribou distinguishes a zone of high connectivity with a high potential conservation value along 70° of longitude. Causes of this pattern are unknown, but our study allowed us to discount habitat variables and climate normals as possible drivers, since these covariates were already included in our model. We propose two hypotheses to explain this spatial pattern: behaviour-driven and history-driven hypotheses. The behaviour-driven hypothesis considers that the actual latent pattern reflects the meta-population structure of the different boreal caribou herds in the region and may depict zones of intensive exchanges amongst populations. The history-driven hypothesis postulates that historical factors associated with range fidelity have shaped the spatial distribution of boreal caribou populations over time and that the actual latent spatial pattern is a relic of this evolution. These two hypotheses could be separated based on their expected spatial patterns, according to the approach advocated by [24]. Our study provides a starting point, but further studies will be needed to validate or refute these hypotheses.

## Supporting Information

Figure S1 **Bar plot showing the frequency of top-ranked models selected over the 1000 random subsamples (20% of the data each).** Red number at the top of each bar represents the rank order of the best model with 100% of the data (see Table 2).
(DOCX)

Figure S2 **Sensitivity analyses regarding the effect of various shape parameters on coefficients of regression (median ± 95% credible interval).** Increasing the value of the shape parameter leads to decreasing smoothness of the spatial random effect. When the shape parameter > 29.56, spatial models (white dots) become equivalent to non-spatial models (black dots). Note that the scale of y-axis differs among plots and that the scale of x-axis is interrupted above 29.
(DOCX)

Table S1 **Loadings of explanatory variables related to conifer stands on the first two dimensions of a Principal Component analysis with covariance matrix across the study area in Québec, Canada.**
(DOCX)

Text S1 **Additional information on statistical models.**
(DOCX)

Text S2 **Model selection and spatial autocorrelation.**
(DOCX)

Text S3 **Inference and residual spatial autocorrelation.**
(DOCX)
