# Correction: Explaining Geographic Gradients in Winter Selection of Landscapes by Boreal Caribou with Implications under Global Changes in Eastern Canada

**DOI:** 10.1371/annotation/9dcd31fb-519a-44f9-a05a-46da149513e9

**Published:** 2013-12-19

**Authors:** Julien Beguin, Eliot J. B. McIntire, Daniel Fortin, Steven G. Cumming, Frédéric Raulier, Pierre Racine, Claude Dussault

In the "Statistical analyses" section, part of Equation 1 was missing. The publisher apologizes for the error. Please view the correct equation here: http://plosone.org/corrections/pone.0078510.e001.cn.tif

