# Supplementary material for: Explaining Geographic Gradients in Winter Selection of Landscapes by Boreal Caribou with Implications under Global Changes in Eastern Canada
Source: PLoS One. 2013 Oct 23;8(10):e78510. doi: 10.1371/journal.pone.0078510 (PMC3806842; doi:10.1371/journal.pone.0078510)

**Figure S1.** Bar plot showing the frequency of top-ranked models selected over the 1000 random subsamples (20% of the data each). Red number at the top of each bar represents the rank order of the best model with 100% of the data (see Table 2).


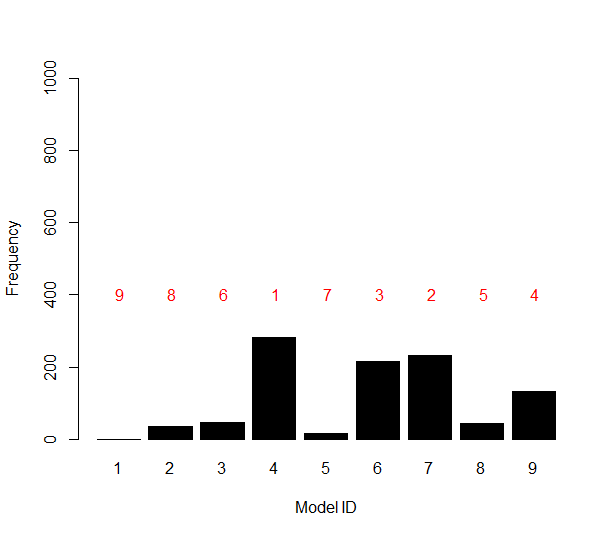

Supplement: Figure S1 — Bar plot showing the frequency of top-ranked models selected over the 1000 random subsamples (20% of the data each). Red number at the top of each bar represents the rank order of the best model with 100% of the data (see Table 2). (DOCX) [file pone.0078510.s001.docx]
