# Supplementary material for: Explaining Geographic Gradients in Winter Selection of Landscapes by Boreal Caribou with Implications under Global Changes in Eastern Canada
Source: PLoS One. 2013 Oct 23;8(10):e78510. doi: 10.1371/journal.pone.0078510 (PMC3806842; doi:10.1371/journal.pone.0078510)

**Figure S2**. Sensitivity analyses regarding the effect of various shape parameters on coefficients of regression (median ± 95% credible interval). Increasing the value of the shape parameter leads to decreasing smoothness of the spatial random effect. When the shape parameter > 29.56, spatial models (white dots) become equivalent to non-spatial models (black dots). Note that the scale of y-axis differs among plots and that the scale of x-axis is interrupted above 29.

**
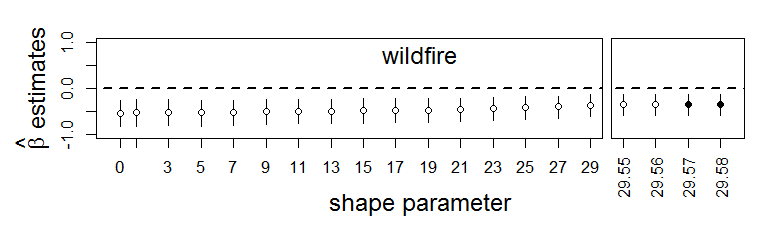
**


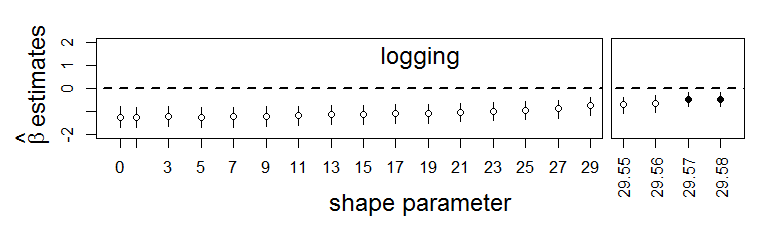


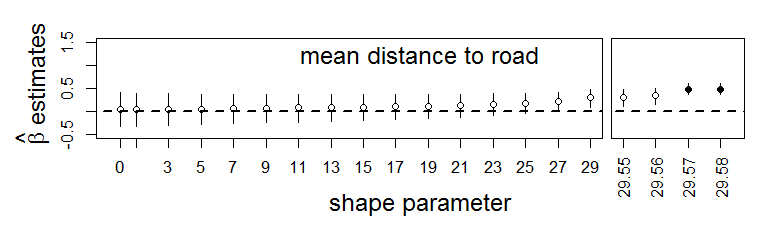


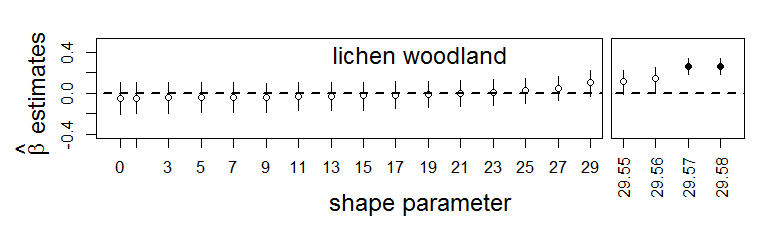


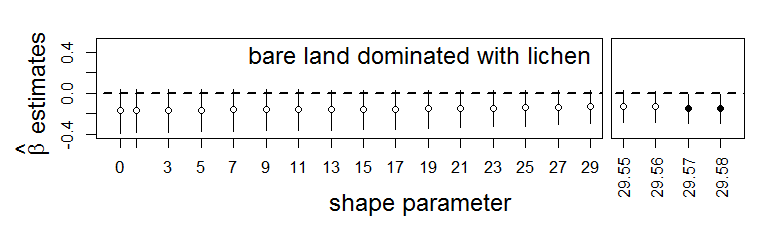


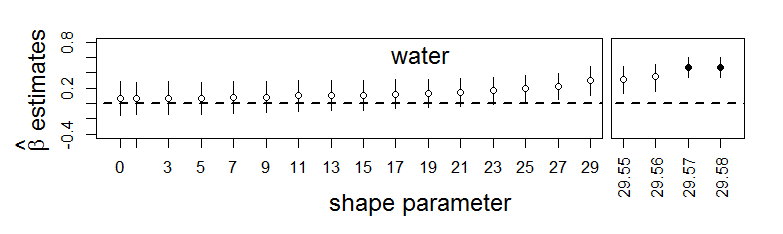


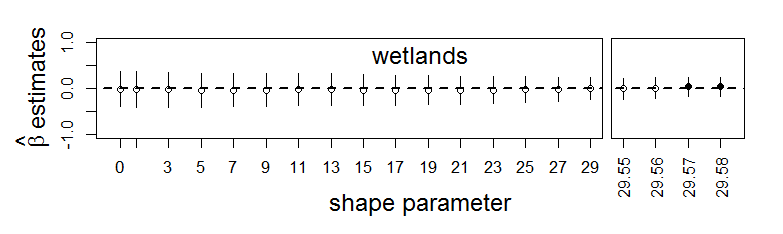


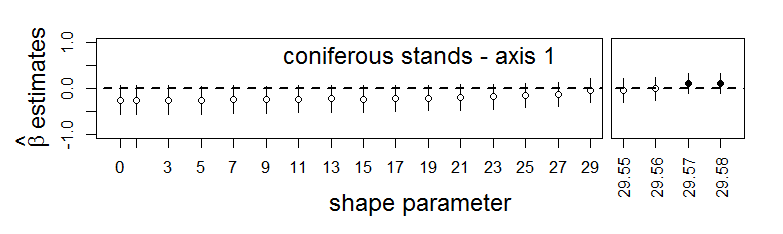


coniferous stands - PC 1


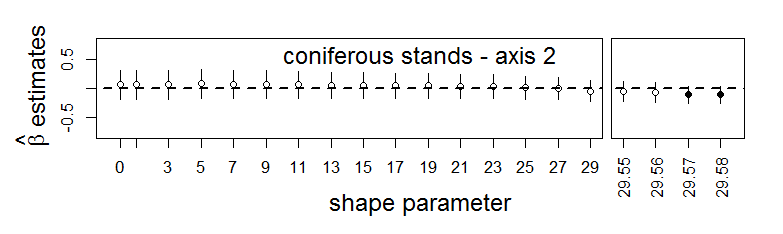


coniferous stands - PC 2


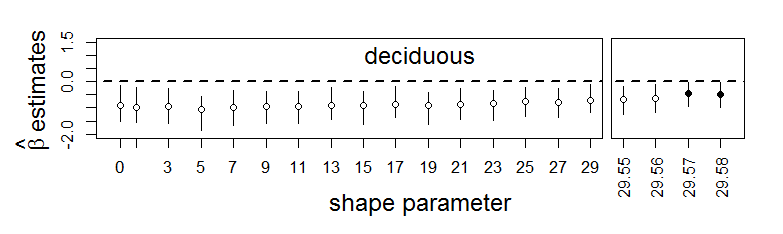


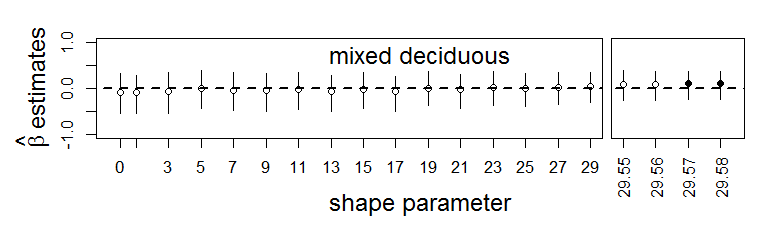

Supplement: Figure S2 — Sensitivity analyses regarding the effect of various shape parameters on coefficients of regression (median ± 95% credible interval). Increasing the value of the shape parameter leads to decreasing smoothness of the spatial random effect. When the shape parameter > 29.56, spatial models (white dots) become equivalent to non-spatial models (black dots). Note that the scale of y-axis differs among plots and that the scale of x-axis is interrupted above 29. (DOCX) [file pone.0078510.s002.docx]
