# Supplementary material for: Explaining Geographic Gradients in Winter Selection of Landscapes by Boreal Caribou with Implications under Global Changes in Eastern Canada
Source: PLoS One. 2013 Oct 23;8(10):e78510. doi: 10.1371/journal.pone.0078510 (PMC3806842; doi:10.1371/journal.pone.0078510)
Supplement: Table S1 — Loadings of explanatory variables related to conifer stands on the first two dimensions of a Principal Component analysis with covariance matrix across the study area in Québec, Canada. (DOCX) [file pone.0078510.s003.docx]

**Table S1.** Loadings of explanatory variables related to conifer stands on the first two dimensions of a Principal Component analysis with covariance matrix across the study area in Québec, Canada.

|  | PCA1 | PCA2 |
| --- | --- | --- |
| black spruce - jack pine | -0.15 | -0.01 |
| black spruce | 0.07 | **-0.99** |
| black spruce - balsam fir | **0.98** | 0.06 |
| balsam fir | 0.09 | 0.07 |
| jack pine | -0.04 | 0.01 |
| mixed resinuous | -0.02 | 0.04 |
| Eigenvalues | 15.56 | 14.85 |
| Percentage variance | 46.93 | 42.74 |
| Cumulative percentage variance | 46.93 | 89.67 |
