# Supplementary material for: Explaining Geographic Gradients in Winter Selection of Landscapes by Boreal Caribou with Implications under Global Changes in Eastern Canada
Source: PLoS One. 2013 Oct 23;8(10):e78510. doi: 10.1371/journal.pone.0078510 (PMC3806842; doi:10.1371/journal.pone.0078510)
Supplement: Text S1 — Additional information on statistical models. (DOCX) [file pone.0078510.s004.docx]

**Text S1. Additional information on statistical models.**

Although we used the same data as Fortin et al. (2008) to document the presence of boreal caribou tracks, our modelling approach and covariates differed markedly from theirs (e.g., their model did not include any climate variables). The use of regular lattice-based models, together with up-to-date forest inventory data, allowed us to obtain new inferences on climate, vegetation attributes, and disturbances. In addition, we used different statistical models with (i) a random intercept model to account for the spatio-temporal structure of inventory aerial blocks imposed by our design and (ii) a spatial random effect, instead of an autocovariate term in a fixed effect autologistic model, to account for spatial autocorrelation. Given the differences in approach between Fortin et al. (2008) and the present study, differences in the results are expected. To ensure, however, that the presence of geographical gradients in the selection of certain habitat classes was present in our data and not simply the result of a statistical artefact, we compared a model with only linear effects of covariates on a logit scale (model 3; Table 2) with a model with the same covariates but containing interaction terms between covariates and latitude/longitude (model 6; Table 2). Interaction terms were selected using a screening process where only significant interaction terms (e.g., credible intervals of parameters not overlapping zero) between covariates and latitude/longitude (in UTM coordinates) were retained using a backwards, stepwise procedure.

**References**

Fortin D, Courtois R, Etcheverry P, Dussault C, Gingras A (2008) Winter selection of landscapes by woodland caribou: behavioural response to geographical gradients in habitat attributes. Journal of Applied Ecology 45: 1392-1400.
