# Supplementary material for: Explaining Geographic Gradients in Winter Selection of Landscapes by Boreal Caribou with Implications under Global Changes in Eastern Canada
Source: PLoS One. 2013 Oct 23;8(10):e78510. doi: 10.1371/journal.pone.0078510 (PMC3806842; doi:10.1371/journal.pone.0078510)
Supplement: Text S2 — Model selection and spatial autocorrelation. (DOCX) [file pone.0078510.s005.docx]

**Text S2. Model selection and spatial autocorrelation.**

Model comparisons and inference can be invalidated by the presence of spatial autocorrelation in model residuals. Bayesian analyses offer a flexible framework for fitting hierarchical spatial models, e.g., spatial GLMM (generalised linear mixed-models), especially since fast and accurate Bayesian approximations are now freely available (Rue *et al.* 2009) and greatly reduce computational demands. Deviance Information Criterion (DIC) is commonly used to compare the fit of alternative Bayesian models under the parsimony principle (Spiegelhalter *et al.* 2002), but theoretical foundations of DIC-comparisons have little support for this class of models (Plummer 2008) and can even lead to poor conclusions when the goal is to compare the importance of different environmental covariates among models. We thus constrained our model selection procedure to non-spatial GLMMs, as described in Equation 1. We verified if spatial autocorrelation had an impact on the model selection procedure. To do so, we randomly selected 20% of cells in the original data set using a stratified sampling scheme with survey blocks as strata. The mean Euclidean distance between sampled cells and their four sampled nearest-neighbours increased from 10 km in the whole data set to ~21 km in data subsets. This intensity of subsampling is expected to remove or reduce spatial auto-correlation in the original data set. We repeated this operation to produce 1000 independent subsamples and then fitted each candidate model to each data subset and calculated the frequencies of the best-ranked models over the 1000 trials. Finally, we compared the frequencies of the best-ranked models over the 1000 trials with the rank of candidate models obtained with 100% of the data. Difference in the model ranking between these two methods would indicate that spatial autocorrelation impacts the model selection procedure with 100% of the data. The graph below (see Figure S1) shows that the rank order of selection frequencies as top models among the 1000 subsamples match almost perfectly the same as the ordering of model DIC in Table 2. We conclude that spatial autocorrelation only had a negligible effect on the result of the model selection procedure.

**References**

Plummer M (2008) Penalized loss functions for Bayesian model comparison. Biostatistics 9: 523-539.

Rue H, Martino S, Chopin N (2009) Approximate Bayesian inference for latent Gaussian models by using integrated nested Laplace approximations. Journal of the Royal Statistical Society Series B-Statistical Methodology 71: 319-392.

Spiegelhalter DJ, Best N, Carlin B, van der Linde A (2002). Bayesian measures of model complexity and fit. Journal of the Royal Statistical Society Series B-Statistical Methodology 64: 583-640.
