# Supplementary material for: Explaining Geographic Gradients in Winter Selection of Landscapes by Boreal Caribou with Implications under Global Changes in Eastern Canada
Source: PLoS One. 2013 Oct 23;8(10):e78510. doi: 10.1371/journal.pone.0078510 (PMC3806842; doi:10.1371/journal.pone.0078510)
Supplement: Text S3 — Inference and residual spatial autocorrelation. (DOCX) [file pone.0078510.s006.docx]

**Text S3. Inference and residual spatial autocorrelation*.***

In addition to model selection and hypothesis tests, we were also interested in making inference on parameters. For inference, we used the complete data set, together with the simplest and top-ranked model that had been selected in the previous step (see model 4 in Table 2). To avoid type-I error caused by residual spatial autocorrelation, we modelled spatial autocorrelation among cells using a Markov representation on a regular grid of a continuous Gaussian field with a Matérn covariance function such as:

$$Corr\left( d \right)\propto\left( \kappa d \right)^{\nu} K_{\nu}(\kappa d)$$

Where $d$ is the Euclidean distance, $K_{v}()$ is the Bessel function of order $\nu$. The range is defined to be $\sqrt{8}/\kappa$ and it is the distance at which two cells are practically uncorrelated. This Matérn function is defined by two hyperparameters: the range (*r*) and the precision (τ). The range defines the distance at which two cells become uncorrelated, while the precision defines the degree of smoothness of the spatial random effect. Based on the variogram of the residuals of the non-spatial GLMM, we fixed the range to be nine times the distance between two neighbouring cells. The Matern covariance function implies a dense covariance matrix that greatly increases computational demands and processing time (Minasny & McBratney 2005). The INLA software uses a Markov representation of the Matérn field which was introduced by Lindgren et al. (2011). The Markov representation offers several computational advantages and greatly reduces the running time to fit such model (see Lindgren et al. 2011). Based on sensitivity analyses (see Figure S2), we assigned the precision a Gamma(shape = 29, scale = 0.001) to avoid overfitting (Beguin *et al.* 2012). A sum-to-zero constraint was applied to the estimation of the spatial random effect to ensure parameter identifiability*.*

**References**

Beguin J, Martino S, Rue H, Cumming SG (2012) Hierarchical analysis of spatially autocorrelated ecological data using integrated nested Laplace approximation. Methods in Ecology and Evolution 3: 921-929.

Lindgren F, Lindstrøm J, Rue H (2011) An explicit link between Gaussian fields and Gaussian Markov random fields: The stochastic partial differential equation approach*.* Journal of the Royal Statistical Society Series B-Statistical Methodology 73: 423-498.

Minasny B, McBratney AB (2005) The Matérn function as a general model for soil variograms. Geoderma 128: 192-207.
